# Clinical Decision Support Systems: Effective Solution for Diagnosis, Treatment, and Management of Patients Affected by Poisoning

**Published:** 2019-07

**Authors:** Zahra MAHMOUDVAND, Shahin SHADNIA, Sharareh ROSTAM NIAKAN, Marjan GHAZISAEEDI

**Affiliations:** 1. Department of Health Information Management, School of Allied Medical Sciences, Tehran University of Medical Sciences, Tehran, Iran; 2. Toxicological Research Center, Department of Clinical Toxicology, Loghman Hakim Hospital, School of Medicine, Shahid Beheshti University of Medical Sciences, Tehran, Iran

## Dear Editor-in-Chief

Poisoning is considered a public health problem in the world. It is a very common cause of hospital admissions and even death worldwide. It has been reported to be the cause of death in as many as 593000 people in the developing world annually (intentional and unintentional) (1). Poisoning is the ninth cause of death among young adults (15 to 29 yr old) (2).

Poisoning requires accurate evaluation and prompt treatment. Time is an important factor in the treatment of poisoning, and a quick and appropriate decision can lead to a better prognosis (3). The decision making to diagnose, manage and follow up the patients affected by verity of poisoning materials are complex. There are a lot of poisoning materials and different care plans and guidelines to manage them. This makes a complexity requiring a system to aid decision makers for optimum option. Actually, health professionals in hospitals who handle such cases do not have enough experience and training for the last edition care plan and guidelines to manage those cases. The implementation of the Clinical Decision Support Systems (CDSS) would help alleviate that problem (4).

CDSS can provide timely information for the diagnosis and treatment of poisoning (5). They have been designed to help directly physicians in decision-making situations. They compare patient’s profile with a computerized clinical database and offer patient-specific suggestions to the physician or patient to make better decisions.

Several decision support systems have been made for the diagnosis and treatment of poisoning. Expert System for Poisoning (ESP) is a clinical decision support system that provides timely information on poisoning. This system allows rapid assessment of the patient’s condition by providing appropriate advice to physicians (4). Antidote Application (AA) is a decision support system in the field of toxicology that offers patient-specific antidote treatment recommendations. This system has the potential to reduce the delay in the prescription of initial dose and drug errors (6). Interactive Poison Expert for Classification and Control (IPECAC) is a specialized system for managing the occurrence of poisoning, which is mostly used in emergency departments and physicians’ clinics (7). SETH is a knowledge-based system for the management of acute drug poisoning in adults. This system provides patient-specific treatment advice (8). The Inreca system works according to the case-based reasoning technology designed to reduce the time of decision-making, especially in emergencies, to help young and inexperienced medical staffs in their decision-making, and to share existing experiences in different places (5).

The toxicology experts working in poisoning centers use this system to confirm their decisions in complicated conditions. They can also increase their knowledge in different areas of toxicology because even the best specialists do not have the necessary knowledge in all areas.

The CDSS help to assess patient status and manage poisoning cases by providing a list of differential diagnoses without having to wait for experts’ advice (3). The use of these systems can improve care, reduce transportation costs, and save time through remote access. These systems also provide accurate, concise, and unique advice based on patient’s specific parameters. By providing different alerts and reminders, they reduce medical errors, improve the quality of patient care.
